# Association of the *MACROD2* rs6110695 A>G polymorphism with an increasing WBC count in a Korean population

**DOI:** 10.1002/iid3.669

**Published:** 2022-06-25

**Authors:** Jihye Yang, Youngmin Han, Jong Ho Lee, Hye Jin Yoo

**Affiliations:** ^1^ Department of Food and Nutrition National Leading Research Laboratory of Clinical Nutrigenetics/Nutrigenomics, College of Human Ecology Yonsei University Seoul Republic of Korea; ^2^ Research Center for Silver Science, Institute of Symbiotic Life‐TECH Yonsei University Seoul Republic of Korea

**Keywords:** chronic diseases, Korean Chip, *MACROD2* gene, rs6110695 A>G polymorphism, WBC count

## Abstract

**Introduction:**

We aimed to find a novel candidate gene related to the white blood cell (WBC) count in a Korean population. Since WBC count has been reported to have a relation to the risk of chronic diseases according to previous literature, WBC level prediction can be helpful for managing future risk of chronic disease development. In this aspect, a gene newly found in the present study is expected to be utilized as a tool for judging an individual's WBC level.

**Methods:**

Based on the 153 study participants' genotype data produced by the Korean Chip. The* mono‐adenosine diphosphate ribosylhydrolase 2 *(*MACROD2*) rs6110695 A>G polymorphism had a significant strong association with WBC count, thus, the *MACROD2* gene emerged as a novel candidate gene for WBC count. To verify the effects of the single‐nucleotide polymorphisms on WBC count, the participants were grouped according to the rs6110695 AA and AG genotypes.

**Results:**

WBC to apolipoprotein A‐I ratio, WBC count, granulocyte to lymphocyte ratio, monocyte to platelet ratio, and interferon‐γ level were significantly higher in the AG genotype group than in the AA genotype group. Through the receiver operating characteristic curve analysis, the rs6110695 AA and AG genotypes were discriminated by the optimal WBC count cutoff value of 5.450. As expected, the results in the participants having a WBC count over 5.450 were similar to the AG genotype group.

**Conclusions:**

We revealed that the *MACROD2* rs6110695 AG genotype has an association with increasing WBC count. Since, as previous literature described, WBC count is one of the main risk factors for chronic diseases, WBC count measurement in individuals with the rs6110695 AG genotype that was found in the present study may help manage future chronic disease risk.

## Graphical abstract

We revealed the *MACROD2* rs6110695 AG genotype has an association with increasing white blood cell (WBC) count, which is one of the risk factors for chronic disease in a Korean population. WBC count measurement in individuals with the rs6110695 AG genotype may help manage the future risk of developing chronic disease.

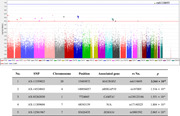

## 1 INTRODUCTION

White blood cells (WBCs) are a well‐known biomarker of systemic inflammation. The WBC count has been reported to be associated with chronic diseases, including cancer, type 2 diabetes, hypertension, and obesity^1^, ^2^, ^3^, ^4^, ^5^, ^6^; our previous study also found a link between WBC count alteration and type 2 diabetes risk.^7^ In addition, various studies have demonstrated that the WBC count is a valuable predictor of all‐cause mortality, especially in elderly individuals.^8^, ^9^, ^10^ Therefore, carefully monitoring WBC counts can be helpful for managing future risk of chronic disease and mortality.

One of the factors that affects an individual's WBC count change is genetic polymorphism. For example, genome‐wide association studies (GWASs) have revealed several single‐nucleotide polymorphisms (SNPs) related to hematological traits, including WBC count.^11^, ^12^ Another study also reported that rs8078723, rs2305482, and rs2302777 are associated with WBC count in the Korean population.^13^ However, the above GWASs involved foreign populations, and the latter study used a genetic analysis tool that is appropriate for Western people. As genetic traits differ among ethnicities, using a proper analysis tool developed for a specific population is essential. Korean Chip (K‐CHIP) is an ideal genetic analysis tool (customized chip) for analyzing SNPs in the Korean population. Thus, the present study used K‐CHIP to identify novel SNPs related to WBC count. The *mono‐adenosine diphosphate* (*ADP*) *ribosylhydrolase 2* (*MACROD2*) rs6110695 A>G polymorphism is an intron variant located at chromosome 20:15623227 (GRCh38.p13) in humans.^14^ The minor allele frequency (MAF) of this SNP (G allele) is 0.080557 in the total global population. In detail, the MAFs in the European and African populations are 0.07579 and 0.1247, respectively. Asians showed a MAF of 0.031, which is lower than other racial populations; in particular, MAF for East Asian is 0.028 and for other Asian is 0.05. In the Korean population, the same ethnic group of the present study, the MAF of the *MACROD2* rs6110695 A>G polymorphism has been reported as 0.0317 in the Korea Genome Project study and 0.0332 in the Korea Reference Genome Database (KRGDB).^14^

The *MACROD2* gene encodes a macrodomain‐containing protein with mono‐ADP ribosylhydrolase activity.^15^ Studies have demonstrated that the *MACROD2* gene is associated with chronic diseases such as cancers, hypertension, and obesity.^16^, ^17^, ^18^, ^19^, ^20^, ^21^, ^22^ Because close relationships have been observed between the risk of chronic diseases and systemic inflammation for which the representative marker is WBC count, there is a potential association between the *MACROD2* gene and WBC count.

Overall, there is a lack of investigation on associations between *MACROD2* genetic polymorphism and WBC count to date. Hence, this study aimed to identify WBC count‐related SNPs using K‐CHIP and to verify whether the *MACROD2* rs6110695 A>G polymorphism is a novel candidate gene related to WBC count in the Korean population.

## 2 MATERIALS AND METHODS

### 2.1 Study population

A total of 197 healthy Korean individuals were recruited for this study at Yonsei University (June 2020 to April 2021). Inclusion criteria were agreeing to participate in the study, Korean and aged 20−80 (male and female), and no severe disease. The exclusion criteria were as follows: a current diagnosis, symptoms, and/or history of myocardial infarction, cerebrovascular disease, neurological disease, mental illness, liver disease, renal disease, pulmonary diseases, cancer including leukemia, autoimmune disease, and other severe immune‐related disease; pregnancy or lactation; and judged by the researchers as being inappropriate for the study. The purpose of this study was carefully explained to all of the participants, and they provided written informed consent. The Institutional Review Board of Yonsei University approved the study protocol, which complied with the Declaration of Helsinki (IRB No.: 7001988‐202107‐BR‐1177‐02).

### 2.2 Blood sample collection

The participants were instructed to fast overnight for at least 12 h before venous blood draw; samples were collected in serum and EDTA‐treated tubes (BD Vacutainer; Becton, Dickinson and Company). The tubes were centrifuged (1200 rpm, 20 min, 4°C) to obtain serum and plasma, and aliquots were stored at −80°C before analysis.

### 2.3 Anthropometric measurement

Weight (UM0703581; Tanita) and height (GL‐150; G‐Tech International) were measured while the participants wore light clothing with shoes removed. Body mass index (BMI) was calculated by dividing weight (kg) by height (m^2^). Waist and hip circumferences (cm) were measured using a plastic measuring tape with measurements to the nearest 0.1 cm at the umbilical level and protruding part, respectively, while the participants were in an upright standing posture. The waist to hip ratio was calculated by dividing waist circumference by hip circumference. Systolic and diastolic blood pressures (BPs) were measured using a random‐zero sphygmomanometer (HM‐1101; Hico Medical Co., Ltd) after the participants rested for at least 10 min in a seated position.

### 2.4 Assessment of biochemical characteristics

For lipid profiles, serum fasting triglyceride, total cholesterol, high‐density lipoprotein (HDL)‐cholesterol, low‐density lipoprotein (LDL)‐cholesterol, apolipoprotein A‐I, and apolipoprotein B levels were assessed. Triglyceride and total cholesterol levels were measured using commercial TG and CHOL kits (Roche), respectively. The HDL‐cholesterol level was measured with HDL‐C Plus kits (Roche), and the resulting color reactions of the assay were analyzed with a Hitachi 7600 autoanalyzer (Hitachi). The LDL‐cholesterol level was calculated using the following equation (the Friedewald formula): LDL‐cholesterol = total cholesterol − (HDL‐cholesterol + [triglyceride ÷ 5]). Apolipoprotein A‐I and apolipoprotein B levels were assessed using the turbidimetric immunoassay method, and the resulting reactions were analyzed using a Cobas 6000 C501 (Roche).

For glucose/insulin resistance‐related markers, serum fasting glucose and insulin levels, homeostatic model assessment for insulin resistance (HOMA‐IR), and plasma adiponectin levels were measured. Glucose was assessed with the hexokinase method, and the resulting color reactions of the assay were analyzed using a Hitachi 7600 autoanalyzer (Hitachi). Insulin levels were assessed with the electrochemiluminescence immunoassay method and a Cobas 6000 E801 (Roche). HOMA‐IR was calculated using the following equation: HOMA‐IR = insulin × glucose ÷ 405. Adiponectin levels were measured via Human Adiponectin ELISA kits (Otsuka Pharmaceutical Co., Ltd), with a VERSA max microplate reader (Molecular Devices) used to evaluate the color reactions of the assay.

### 2.5 Total blood cell count and inflammatory marker assessment

WBC (lymphocytes, monocytes, and granulocytes) counts and the percentage of each component as well as platelet counts were measured using a HORIBA ABX diagnostic analyzer (HORIBA ABX SAS; Parc Euromedicine). Using these counts, the WBC to apolipoprotein A‐I ratio, monocyte to lymphocyte ratio (MLR), granulocyte to lymphocyte ratio (GLR), platelet to lymphocyte ratio (PLR), and monocyte to platelet ratio (MPR) were calculated.

High‐sensitivity C‐reactive protein (hs‐CRP), interleukin (IL)‐1β, IL‐2, IL‐6, IL‐12, tumor necrosis factor (TNF)‐α, and interferon (IFN)‐γ were measured as inflammatory markers. The hs‐CRP level was assessed with CRPHS reagent kits (Roche) and a Cobas C502 (Roche). IL‐1β, IL‐6, and TNF‐α levels were determined using Bio‐Plex Reagent kits (Bio‐Rad Laboratories) and a Luminex 200 (Luminex Corporation). IL‐2, IL‐12, and IFN‐γ levels were measured with Human IL‐2 ELISA kits (Cusabio Biotech), High Sensitivity Human IL‐12 (p70) ELISA kits (Genway Biotech Inc.), and IFN‐γ High Sensitivity Human ELISA kits (Abcam plc), respectively; the absorbance at 450 nm of the resulting reactions of the assays were evaluated using a Victor×5 2030 Multilabel Plate Reader (PerkinElmer).

### 2.6 SNP genotyping array

All equipment and resources required for Axiom 2.0 Assays with automated target preparation are available in Axiom 2.0 Assay Automated Workflow User Guide (P/N 702963). Using the Axiom 2.0 Reagent kits (P/N 901758), approximately 200 ng of genomic deoxyribonucleic acid (DNA) was amplified and randomly fragmented into 25−125 bp fragments. A fragmentation step then further reduced the amplified products to segments of approximately 25−50 bp, which were then end‐labeled using biotinylated nucleotides. The samples were denatured and transferred to the hybridization tray to begin hybridization in GeneTitan MC Instrument (Affymetrix). The hybridization step followed the GeneTitan Multichannel Instrument User's Manual (P/N 08‐0306) using Axiom BiobankPlus Genotyping Array KNIHv1.1. After ligation, the arrays were stained and imaged using GeneTitan MC Instrument (Affymetrix), and images were analyzed using Affymetrix GeneChip Command Console Software User Manual (P/N 702569). Genotype data were produced using the K‐CHIP available through the K‐CHIP consortium. K‐CHIP was designed by the Center for Genome Science, Korea National Institute of Health, Republic of Korea (4845‐301, 3000‐3031).

Samples that showed the following inclusion thresholds were excluded (quality control): sex inconsistency, markers with a high missing rate >0.05, individuals with a high missing rate >0.1, a MAF <0.01, and significant deviation from Hardy−Weinberg equilibrium (*p* <1 × 10^−6^). Additionally, SNPs related to one another in linkage disequilibrium were excluded. The remaining 356,375 SNPs and 153 participants were included in the subsequent association analysis.

### 2.7 Statistical analysis

PLINK ver. 1.07 (http://zzz.bwh.harvard.edu/plink/) was used for quality control of SNP data and for assessing associations between WBC counts and SNPs via linear regression analysis with adjustment for age, sex, and BMI. A Manhattan plot was produced by Haploview 4.2 (http://www.broadinstitute.org/haploview/haploview). SPSS ver. 26.0 (IBM) was used for other statistical analyses. To find an optimal WBC count cutoff value that best discriminates rs6110695 genotypes, receiver operating characteristic (ROC) curve analysis with Youden's index calculation was performed. For between‐group comparisons (between genotypes AA vs. AG; between WBC count cutoff values WBC <5.450 vs. WBC ≥5.450), normally distributed data or data that followed a normal distribution after logarithmic transformation were analyzed by independent *t*‐tests; non‐normal distributed data even after logarithmic transformation were analyzed by the Mann−Whitney *U* tests. In the case of between‐group comparison of the WBC count cutoff value, analysis of covariance tests were used to adjust for age, sex, and weight. These data are presented as the mean ± standard error) for descriptive purposes. Sex distribution was analyzed using *χ* ^2^ or Fisher's exact tests, and the data are presented as numbers and percentages. Lastly, Pearson's correlation analysis was conducted to confirm a relationship between some variables. All *p* < 0.05 (two‐tailed), but *p* < 1 × 10^−8^ for SNPs, were considered to be significant.

## 3 RESULTS

**Figure 1:**
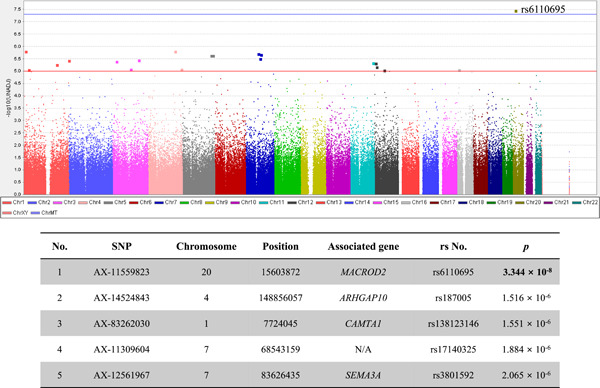
Manhattan plot and top five SNPs associated with WBCs. The blue line indicates a significant level (*p* = 5 × 10^−8^). The red line indicates a suggestive level (*p* = 1 × 10^−5^). SNPs, single‐nucleotide polymorphisms; WBCs, white blood cell.

A total of 356,375 SNPs and 153 participants were included in the final analysis. A Manhattan plot and the top five SNPs that showed associations with WBC count are provided in Figure 1. The SNP most strongly associated with WBC count was rs6110695 in the *MACROD2* gene (*MACROD2* rs6110695 A>G polymorphism). Known information on the MAF of rs6110695 in the Korean population is G = 0.0317 and G = 0.0332 from the Korean Genome Project study and the KRGDB study, respectively.^14^ Indeed, the MAF among all participants in the present study was G = 0.0392. Unfortunately, no individuals with minor allele homozygosity (GG genotype) were found in our population; therefore, comparisons by genotype were carried out between major allele homozygotes (AA genotype) and heterozygotes (AG genotype).

### 3.1 Analyses of clinical/biochemical characteristics and total blood cell count/inflammatory markers according to rs6110695 genotype

The study participants (*n* = 153) were divided into an rs6110695 AA genotype group (*n* = 141) and rs6110695 AG genotype group (*n* = 12). Table 1 shows the clinical and biochemical characteristics of the two groups. The total cholesterol level and WBC to apolipoprotein A‐I ratio were significantly lower and higher in the AG than in the AA genotype group, respectively. No significant differences between the groups were found for the other parameters (Table 1).

**Table 1:** Clinical and biochemical characteristics in the study participants according to rs6110695 genotype

	Total (*n* = 153)		*p*
	AA genotype (*n* = 141)	AG genotype (*n* = 12)	
Age (year)^b^	52.6 ± 0.85	48.2 ± 2.92	0.124
Male/female *n*, (%)	18 (12.8)/123 (87.2)	1 (8.3)/11 (91.7)	1.000
Weight (kg)^a^	60.4 ± 0.70	62.4 ± 1.74	0.348
BMI (kg/m^2^)	23.8 ± 0.22	23.7 ± 0.74	0.836
Waist (cm)	85.9 ± 0.59	85.4 ± 2.55	0.789
Waist to hip ratio	0.90 ± 0.00	0.88 ± 0.02	0.238
Systolic BP (mmHg)	118.0 ± 1.21	118.4 ± 3.35	0.926
Diastolic BP (mmHg)	74.0 ± 0.87	73.6 ± 1.84	0.894
Triglyceride (mg/dl)^a^	124.2 ± 5.26	109.1 ± 19.2	0.294
Total cholesterol (mg/dl)	210.7 ± 2.66	191.1 ± 11.2	**0.045**
HDL‐cholesterol (mg/dl)^a^	57.2 ± 1.32	54.8 ± 4.65	0.547
LDL‐cholesterol (mg/dl)	126.7 ± 2.90	114.5 ± 13.2	0.225
Apolipoprotein A‐I (mg/dl)^a^	157.5 ± 2.17	149.7 ± 7.03	0.301
Apolipoprotein B (mg/dl)^a^	107.0 ± 2.09	96.0 ± 9.78	0.219
WBC to apolipoprotein A‐I ratio^a^	0.03 ± 0.00	0.05 ± 0.00	**<0.001**
Glucose (mg/dl)^a^	92.0 ± 0.84	89.8 ± 2.56	0.459
Insulin (μIU/ml)^a^	9.20 ± 0.41	9.87 ± 1.79	0.813
HOMA‐IR^a^	2.10 ± 0.10	2.18 ± 0.41	0.950
Adiponectin (ng/ml)^b^	8.24 ± 0.42	8.52 ± 1.50	0.914

*Note*: All *p *< 0.05 were considered to be significant (shown in bold). Mean ± standard error.

Abbreviations: BMI, body mass index; BP, blood pressure; HDL, high‐density lipoprotein; HOMA‐IR, homeostatic model assessment‐insulin resistance; LDL, low‐density lipoprotein; WBC, white blood cell.

*p* Values of continuous variables were derived from independent *t*‐tests. Sex distribution was tested by Fisher's exact test.

^a^ Variables were tested by nonparametric tests (Mann−Whitney *U* tests).

^b^ Variables tested following logarithmic transformation.

Table 2 presents the results of total blood cell count and inflammatory marker analyses between the two groups. Regarding the total blood cell count, the WBC count, monocyte count, granulocyte count, granulocyte percentage, GLR, and MPR were significantly increased in the AG genotype group compared to the AA genotype group. The MLR also showed a tendency toward increase in the AG genotype group. On the other hand, the lymphocyte percentage was significantly lower in the AG genotype group than in the AA genotype group (Table 2). With regard to inflammatory markers, TNF‐α and IFN‐γ levels showed a significant decrease and increase in the AG genotype group compared to the AA genotype group, respectively. Other inflammatory markers, including hs‐CRP, IL‐1β, IL‐2, IL‐6, and IL‐12 levels, were not significantly different between the two groups (Table 2).

**Table 2:** Total blood cell count and inflammatory markers in the study participants according to rs6110695 genotype

	Total (*n* = 153)		*p*
	AA genotype (*n* = 141)	AG genotype (*n* = 12)	
Total blood cell count			
WBC (×10^3^/μl)^a^	4.97 ± 0.09	6.92 ± 0.40	**<0.001**
Lymphocyte count (×10^3^/μl)^b^	1.78 ± 0.04	2.07 ± 0.13	0.059
Monocyte count (×10^3^/μl)^b^	0.32 ± 0.02	0.49 ± 0.09	**0.010**
Granulocyte count (×10^3^/μl)^a^	2.87 ± 0.07	4.36 ± 0.29	**<0.001**
Lymphocyte (%)	37.3 ± 0.060	30.9 ± 1.67	**0.002**
Monocyte (%)^b^	7.45 ± 0.32	7.49 ± 0.91	0.722
Granulocyte (%)	55.2 ± 0.75	61.7 ± 1.94	**0.016**
Platelet (×10^3^/μl)^b^	243.5 ± 5.88	257.9 ± 11.6	0.154
MLR^a^	0.18 ± 0.01	0.23 ± 0.03	0.054
GLR^a^	1.69 ± 0.05	2.20 ± 0.21	**0.008**
PLR^a^	142.0 ± 3.45	129.6 ± 9.11	0.350
MPR^a^	0.0013 ± 0.00	0.0020 ± 0.00	**0.028**
Inflammatory markers			
hs‐CRP (mg/L)^b^	0.64 ± 0.04	1.37 ± 0.44	0.238
IL‐1β (pg/ml)^b^	0.29 ± 0.03	0.30 ± 0.08	0.554
IL‐2 (pg/ml)^b^	49.0 ± 2.27	47.3 ± 2.22	0.790
IL‐6 (pg/ml)^b^	3.65 ± 0.29	1.33 ± 0.14	0.093
IL‐12 (pg/ml)^b^	4.83 ± 0.32	6.80 ± 1.04	0.114
TNF‐α (pg/ml)^b^	3.21 ± 0.29	0.54 ± 0.01	**0.015**
IFN‐γ (pg/ml)^b^	2.59 ± 0.15	5.71 ± 0.98	**0.002**

Note: All *p *< 0.05 were considered to be significant (shown in bold). Mean ± standard error.

Abbreviations: GLR, granulocyte to lymphocyte ratio; hs‐CRP, high‐sensitivity C‐reactive protein; IFN, interferon; IL, interleukin; MLR, monocyte to lymphocyte ratio; MPR, monocyte to platelet ratio; PLR, platelet to lymphocyte ratio; TNF, tumor necrosis factor; WBC, white blood cell.

*p* Values of continuous variables were derived from independent *t*‐tests.

^a^ Variables tested following logarithmic transformation.

^b^ Variables were tested by nonparametric tests (Mann−Whitney *U* tests).

### 3.2 Optimal cutoff value for WBC count

**Figure 2:**
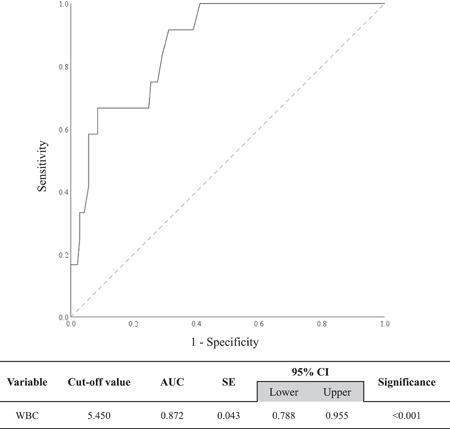
Optimal cutoff value of WBC count to estimate rs6110695 genotypes. AUC, area under the curve; CI, confidence interval; SE, standard error; WBCs, white blood cell.

It was difficult to group the study participants according to WBC count (e.g., abnormally high WBC count group, abnormally low WBC count group, or normal WBC count group) because our study participants mostly had WBC count within the normal range. Therefore, as rs6110695 was found to have a strong linear association with WBC count, a WBC count threshold that best discriminates rs6110695 genotypes was determined by performing ROC curve analysis; it was then used as a basis for dividing the study participants. The optimal cutoff value of WBC count was 5.450 (Figure 2), with sensitivity and specificity of 0.917 and 0.688, respectively (data not shown). In other words, those having a WBC count over 5.450 were more likely to have the AG genotype than the AA genotype of the rs6110695 A>G polymorphism. The area under the curve was 0.872 (95% confidence interval: 0.778−0.955; *p* < 0.001). Thus, WBC count can be considered to have significant discriminating ability in distinguishing rs6110695 genotype (Figure 2).

### 3.3 Analyses of clinical/biochemical characteristics and total blood cell count/inflammatory markers according to WBC cutoff value

Supporting Information: Table 1 shows the clinical and biochemical characteristics of the WBC <5.450 and WBC ≥5.450 groups. The participants in the WBC ≥5.450 group were younger and heavier than those in the WBC <5.450 group; sex distribution also differed significantly. Therefore, age, weight, and sex were adjusted for as confounding factors; statistical significance for the WBC to apolipoprotein A‐I ratio and insulin level remained after thadjustment, which were increased in the WBC ≥5.450 group. HOMA‐IR was higher in the WBC ≥5.450 group than in the WBC <5.450 group; however, its significance disappeared after adjustment. The glucose level tended to decrease in the WBC ≥5.450 group after confounding factor adjustment (Supporting Information: Table 1).

Additionally, the total blood cell count (Supporting Information: Table 2), WBC count, lymphocyte count, monocyte count, granulocyte count, granulocyte percentage, platelet count, and GLR were significantly increased in the WBC ≥5.450 group compared to the WBC <5.450 group before and after adjusting for confounding factors. The MPR was significantly higher in the WBC ≥5.450 group than in the WBC <5.450 group before adjusting; however, an increasing tendency was shown after adjustment. Meanwhile, the lymphocyte percentage was significantly decreased in the WBC ≥5.450 group compared to that in the WBC <5.450 group before and after confounding factor adjustment (Supporting Information: Table 2). In inflammatory marker analysis, IL‐12 and IFN‐γ levels were significantly increased in the WBC ≥5.450 group before the adjustment, though such significance only remained for the IFN‐γ level after adjustment, and the IL‐12 level showed an increasing tendency after adjustment (Supporting Information: Table 2).

### 3.4 Pearson's correlation analysis in the whole study population

As shown in Table 1, WBC to apolipoprotein A‐I ratio was significantly increased in the AG genotype group; and this might be due to an increase in WBC count and a decrease in apolipoprotein A‐I. However, apolipoprotein A‐I in the AG genotype group decreased without statistical significance. Meanwhile, HDL‐cholesterol also decreased in the AG genotype group. Thus, we performed a correlation analysis between apolipoprotein A‐I and HDL‐cholesterol to determine their relationship based on the fact that apolipoprotein A‐I is a major component of HDL‐cholesterol. In Table 3, a significant strong positive correlation between the two variables was shown (*r* = 0.792, *p* < 0.001) as expected. Additionally, using GLR and MPR, which showed statistical significance in Table 2 as blood cell count ratio, we tested the correlation with WBC to apolipoprotein A‐I ratio. As a result, both GLR and MPR showed significant positive correlations with WBC to apolipoprotein A‐I ratio (Table 3). Finally, IFN‐γ, an inflammatory marker increased in the AG genotype group, was used for correlation analysis with insulin and HOMA‐IR. IFN‐γ showed a significant positive correlation with insulin and showed a positively correlated tendency with HOMA‐IR (Table 3).

**Table 3:** Pearson's correlation analysis

	Total (*n* = 153)	
Variables	***r***	***p***
Apolipoprotein A‐I		
HDL‐cholesterol	0.792	<0.001
WBC to apolipoprotein A‐I ratio		
GLR	0.210	0.009
MPR	0.383	<0.001
IFN‐γ		
Insulin	0.191	0.042
HOMA‐IR	0.175	0.063

Abbreviations: GLR, granulocyte to lymphocyte ratio; HDL, high‐density lipoprotein; HOMA‐IR, homeostatic model assessment‐insulin resistance; IFN, interferon; MPR, monocyte to platelet ratio; WBC, white blood cell.

## 4 DISCUSSION

The main finding of the present study is that the WBC count is significantly associated with the *MACROD2* rs6110695 A>G polymorphism and is higher in individuals who carry the rs6110695 AG genotype. Moreover, a high granulocyte count and percentage, WBC to apolipoprotein A‐I ratio, GLR, MPR, and IFN‐γ level were observed in the rs6110695 AG genotype group.

ADP ribosylation is an essential reversible posttranslational protein modification involved in regulating DNA repair, apoptosis, cell proliferation, stress, and immune responses.^23^ Levels of ADP ribosylation are determined by the activities of ADP ribosyltransferases and ADP ribosylhydrolases. MACROD2, which contains a macrodomain for ADP‐ribose recognition, plays a role in sensing and hydrolysis of ADP‐ribose of acidic residues in ribosylated proteins.^23^, ^24^ Therefore, polymorphism of the gene that encodes MACROD2 may lead to an abnormal function of this protein. Indeed, studies consistently demonstrate associations between SNPs in the *MACROD2* gene region and various diseases, including not only chronic diseases (cancer, hypertension, and obesity) but also neurological disorders (autism and Kabuki syndrome).^16^, ^17^, ^18^, ^19^, ^20^, ^21^, ^22^, ^25^, ^26^ The present study revealed the *MACROD2* rs6110695 A>G polymorphism is associated with WBC count that is related to the risk of chronic disease. Altered MACROD2 function induced by this SNP may affect the generation of WBCs, thereby increasing their count.

WBCs comprise lymphocytes, monocytes, and granulocytes. There are three granulocyte subtypes, neutrophils, basophils, and eosinophils, with neutrophils being the most abundant subtype. Among these blood cells, *MACROD2* mRNA expression is higher in neutrophils^27^; in other words, increased expression of *MACROD2* mRNA likely occurs in granulocytes. In the present study, the AG genotype group had a significantly higher WBC count, granulocyte count, and granulocyte percentage than the AA genotype group (Table 2), indicating that granulocyte generation may be accelerated in individuals harboring the minor allele of rs6110695. Note that, however, a minuscule fraction of *MACROD2* and its mRNA are expressed in the bone marrow and blood cells compared to other tissues or cells in the body.^28^

Noonan et al. demonstrated that the knock‐out of the *MACROD2* gene reduces the level of vascular adhesion protein‐1 (VAP‐1), which is expressed on the cell surface of adipose tissue and vascular endothelium and is secreted into the circulation.^29^ VAP‐1 has been reported to induce inflammation, leading to diabetes, hypertension, atherosclerosis, and so forth by involving immune cell trafficking.^29^, ^30^ Thus, it is possible that the increased WBC count in the AG genotype group of the present study might be due to increased inflammation accompanied by increased VAP‐1, which may have been caused by the *MACROD2* rs6110695 mutation. However, this speculation should be approached with caution because this study did not actually measure the association between *MACROD2* rs6110695 A>G polymorphism and VAP‐1 level. Nevertheless, this can give researchers inspiration for further study in figuring out the role of the rs6110695 variant.

Furthermore, total cholesterol in the AG genotype group was significantly decreased, and both HDL‐ and LDL‐cholesterol showed reduced values (but not significant) in the AG genotype group (Table 1). In particular, despite not being significant, a decrease in apolipoprotein A‐I levels appears to be a phenomenon accompanying lowered HDL‐cholesterol; a positive correlation was observed between apolipoprotein A‐I and HDL‐cholesterol (*r* = 0.792, *p* < 0.001) (Table 3). Due to the rising WBC count and reduced apolipoprotein A‐I, the WBC to apolipoprotein A‐I ratio was significantly increased in the AG genotype group (Table 1). Although the underlying mechanism of lipid profile alterations with the *MACROD2* rs6110695 A>G polymorphism cannot be elucidated by our findings, a previous study reported that a high WBC to apolipoprotein A‐I ratio is significantly related to long‐term adverse prognosis in CVD.^31^ Similarly, the neutrophil to apolipoprotein A‐I ratio was identified as a novel predictor for overall survival in patients with hepatocellular carcinoma.^32^ Maintenance of a high WBC to apolipoprotein A‐I ratio seems to be related to poor prognosis of diseases; hence, having the rs6110695 AG genotype is somewhat unfavorable.

Overall, the WBC to apolipoprotein A‐I ratio showed significant positive correlations with GLR (*r* = 0.210, *p* = 0.009) and MPR (*r* = 0.383, *p* < 0.001) in all the total study population (Table 3). As described previously, the neutrophil is the most abundant subtype of granulocyte, thus, GLR can be inferred as the neutrophil to lymphocyte ratio (NLR). Studies have studied NLR to verify its value as a prognostic biomarker for mortality and diseases. A recent study has reported that NLR is associated with overall mortality in the general population in the United States.^33^ In the Asian population, NLR showed a significant association with prevalent chronic conditions.^34^ Additionally, a study has demonstrated that NLR is a good predictor and prognostic marker for metabolic syndrome.^35^ Regarding MPR, only one study has reported high MPR as a potential biomarker for chronic hepatitis C virus infection.^36^ Several studies on monocyte‐platelet interactions resulting in the production of monocyte‐platelet aggregates have been reported. Monocyte‐platelet complexes play a vital role in chronic inflammation, cardiovascular disease, chronic heart disease, and various thrombotic events and are increased in patients with hypertension or diabetes whose atherothrombotic risk is high.^37^, ^38^, ^39^ As monocytes are a substrate of activated platelets, which form complexes, we presumed that a high MPR would implicate a significant chance of monocyte‐platelet complex formation, though markers for monocyte‐platelet complexes were not measured in this study. Taken together, maintaining high GLR and MPR is related to the risk of various non‐health conditions; and even, the AG genotype group in our study exhibited statistically significantly high GLR and MPR. Thus, our data show consistent results that the *MACROD2* rs6110695 A>G polymorphism is disadvantageous to health.

Finally, the AG genotype group in the present study showed significantly increased levels of IFN‐γ, a proinflammatory cytokine that plays a vital role in immune responses. It activates cytotoxic immune cells by stimulating antigen‐presentation to protect against foreign antigens.^40^ Nonetheless, high levels of IFN‐γ contribute to pathological processes such as increased insulin resistance in adipocytes, excessive inflammation, autoinflammation, autoimmunity, and increased tissue damage.^41^ Indeed, in our study, the WBC ≥5.450 group showed not only a significant increase in IFN‐γ levels (Supporting Information: Table S2) but also increased insulin levels and HOMA‐IR which are related to the risk of insulin resistance as one of the chronic disease risks, although the significance of HOMA‐IR disappeared after adjustment for age, sex, and weight (Supporting Information: Table S1). Moreover, IFN‐γ levels had a significant positive correlation with insulin levels and a positively correlated tendency with HOMA‐IR (Table 3). The results imply that individuals with the *MACROD2* rs6110695 AG genotype having a higher WBC count than those with the AA genotype may have an increased chance of developing chronic diseases by elevated IFN‐γ levels.

This study has several limitations. First, the sample size was too small to support statistical power. Second, replication of the results in an independent population could not be performed because of a lack of resources (e.g., time, funding, and the pool of study participants). Third, the exact mechanisms by which this polymorphism interacts with WBC count increases cannot be elucidated based on our data, and previously published literature was used to support our results. Accordingly, further research using a larger population with in‐depth experiments is needed to address these limitations.

## 5 CONCLUSION

Nevertheless, to the best of our knowledge, the clinical relevance of this polymorphism, rs6110695, has never been reported thus far. This is the first study investigating an association between an SNP in the *MACROD2* gene and WBC count; and we revealed that the *MACROD2* rs6110695 AG genotype is related to WBC count increase. In the research field, the results provide basic knowledge and new insight into the relationship between the *MACROD2* rs6110695 A>G polymorphism and WBC count. In the medical field, moreover, as the WBC count is one of the risk factors for chronic disease, checking for the rs6110695 genotype and detecting the WBC count in individuals with the rs6110695 AG genotype can be used as a prediction tool for managing the future risk of developing chronic diseases. Besides, as therapeutic manipulation of ADP‐ribosylation has been reported to positively affect several metabolic disorders,^23^, ^42^ the findings on the polymorphism of the *MACROD2* gene encoding the ADP‐ribosylhydrolase of our study could be the basis for discovering candidate targets for the management and treatment of metabolic disease.

## AUTHOR CONTRIBUTIONS

Jihye Yang conducted the experimental analyses, interpreted the data, and wrote the manuscript draft. Youngmin Han interpreted the data and edited and revised the manuscript. Jong Ho Lee designed the study and provided the experimental setup and samples. Hye Jin Yoo designed the study, performed the statistical analyses, interpreted the data, edited and revised the manuscript, and provided funding. All authors carefully reviewed the final manuscript and approved it for publication.

## CONFLICT OF INTEREST

The authors declare no conflict of interest.

## Supporting information

Supporting information.

Supporting information.

## Data Availability

The data that support the findings of this study are available from the corresponding author upon reasonable request.
