# Supplementary material for: Association of the MACROD2 rs6110695 A>G polymorphism with an increasing WBC count in a Korean population
Source: Immun Inflamm Dis. 2022 Jun 25;10(7):e669. doi: 10.1002/iid3.669 (PMC9233196; doi:10.1002/iid3.669)
Supplement: Supplementary file 1 — Supporting information. [file IID3-10-e669-s001.docx]

**Table S1. Clinical and biochemical characteristics in the study participants according to the WBC cut-off value.**

|  | **Total (*n*=153)** | | | | ***p^a^*** | ***p^b^*** |
| --- | --- | --- | --- | --- | --- | --- |
|  | **WBC <5.450 (*n*=98)** | | **WBC ≥5.450 (*n*=55)** | |  |  |
| Age (year) | 54.1 | ±0.88 | 49.1 | ±1.58 | **0.008** | **-** |
| Male/Female *n*, (%) | 6 (6.10) / 92 (93.9) | | 13 (23.6) / 42 (76.4) | | **0.002** | - |
| Weight (kg)*^†^* | 59.6 | ±0.80 | 62.3 | ±1.11 | **0.045** | - |
| BMI (kg/m^2^)*^†^* | 23.8 | ±0.26 | 24.0 | ±0.35 | 0.674 | 0.887 |
| Waist (cm) | 85.8 | ±0.73 | 86.2 | ±0.95 | 0.743 | 0.961 |
| Waist to hip ratio | 0.90 | ±0.01 | 0.89 | ±0.01 | 0.734 | 0.966 |
| Systolic BP (mmHg) | 117.4 | ±1.47 | 119.2 | ±1.82 | 0.447 | 0.337 |
| Diastolic BP (mmHg) | 73.6 | ±1.05 | 74.7 | ±1.28 | 0.524 | 0.433 |
| Triglyceride (mg/dL)*^†^* | 115.4 | ±5.53 | 128.9 | ±8.74 | 0.205 | 0.179 |
| Total-cholesterol (mg/dL) | 212.2 | ±3.29 | 203.6 | ±4.29 | 0.115 | 0.217 |
| HDL-cholesterol (mg/dL)*^†^* | 58.1 | ±1.56 | 55.0 | ±2.15 | 0.185 | 0.193 |
| LDL-cholesterol (mg/dL) | 128.6 | ±3.45 | 120.6 | ±5.03 | 0.181 | 0.259 |
| Apolipoprotein A-I (mg/dL)*^†^* | 158.5 | ±2.54 | 153.9 | ±3.59 | 0.230 | 0.109 |
| Apolipoprotein B (mg/dL) | 108.1 | ±2.68 | 102.6 | ±3.22 | 0.211 | 0.237 |
| WBC to apolipoprotein A-I ratio*^†^* | 0.03 | ±0.00 | 0.04 | ±0.00 | **<0.001** | **<0.001** |
| Glucose (mg/dL)*^†^* | 92.8 | ±1.02 | 90.1 | ±1.28 | 0.098 | 0.052 |
| Insulin (μIU/mL)*^†^* | 8.52 | ±0.47 | 10.6 | ±0.70 | **0.003** | **0.039** |
| HOMA-IR*^†^* | 1.97 | ±0.12 | 2.35 | ±0.16 | **0.015** | 0.127 |
| Adiponectin (ng/mL) | 8.58 | ±0.50 | 7.31 | ±0.58 | 0.112 | 0.365 |

Mean ± standard error (SE). *^†^* variables tested following logarithmic transformation. *p^a^*-values of continuous variables were derived from independent *t*-tests. *p^a^*-values of the sex distribution were derived from *Chi-squared* tests. *p^b^*-values were derived from *ANCOVA* tests for adjusting age, sex, and weight. All *p*<0.05 were considered to be significant. BMI: body mass index. BP: blood pressure. HDL: high-density lipoprotein. HOMA-IR: homeostatic model assessment-insulin resistance. LDL: low-density lipoprotein. WBC: white blood cell.
