# Supplementary material for: Association of the MACROD2 rs6110695 A>G polymorphism with an increasing WBC count in a Korean population
Source: Immun Inflamm Dis. 2022 Jun 25;10(7):e669. doi: 10.1002/iid3.669 (PMC9233196; doi:10.1002/iid3.669)
Supplement: Supplementary file 2 — Supporting information. [file IID3-10-e669-s002.docx]

**Table S2. Total blood cell count and inflammatory markers in the study participants according to the WBC cut-off value.**

|  | **Total (*n*=153)** | | | | ***p^a^*** | ***p^b^*** |
| --- | --- | --- | --- | --- | --- | --- |
|  | **WBC <5.450 (*n*=98)** | | **WBC ≥5.450 (*n*=55)** | |  |  |
| **Total blood cell count** |  |  |  |  |  |  |
| WBC (×10^3^/μL) | 4.38 | ±0.07 | 6.44 | ±0.12 | **<0.001** | **<0.001** |
| Lymphocyte count (×10^3^/μL) | 1.65 | ±0.04 | 2.08 | ±0.06 | **<0.001** | **<0.001** |
| Monocyte count (×10^3^/μL) | 0.28 | ±0.02 | 0.42 | ±0.04 | **0.002** | **<0.001** |
| Granulocyte count (×10^3^/μL) | 2.45 | ±0.05 | 3.94 | ±0.11 | **<0.001** | **<0.001** |
| Lymphocyte (%) | 38.8 | ±0.68 | 33.4 | ±0.90 | **<0.001** | **<0.001** |
| Monocyte (%) | 7.57 | ±0.36 | 7.25 | ±0.55 | 0.608 | 0.889 |
| Granulocyte (%) | 53.7 | ±0.85 | 59.4 | ±1.17 | **<0.001** | **<0.001** |
| Platelet (×10^3^/μL) | 229.5 | ±5.46 | 271.6 | ±11.0 | **<0.001** | **<0.001** |
| MLR*^†^* | 0.17 | ±0.01 | 0.20 | ±0.02 | 0.180 | 0.107 |
| GLR*^†^* | 1.58 | ±0.06 | 2.00 | ±0.09 | **<0.001** | **<0.001** |
| PLR*^†^* | 145.0 | ±4.15 | 134.0 | ±5.18 | 0.079 | 0.360 |
| MPR*^†^* | 0.0012 | ±0.00 | 0.0016 | ±0.00 | **0.039** | 0.057 |
| **Inflammatory markers** |  |  |  |  |  |  |
| hs-CRP (mg/L) | 0.64 | ±0.05 | 0.67 | ±0.08 | 0.746 | 0.565 |
| IL-1β (pg/mL) | 0.32 | ±0.04 | 0.45 | ±0.06 | 0.083 | 0.396 |
| IL-2 (pg/mL) | 49.4 | ±3.20 | 54.8 | ±3.63 | 0.286 | 0.671 |
| IL-6 (pg/mL) | 4.47 | ±0.48 | 5.67 | ±0.86 | 0.227 | 0.805 |
| IL-12 (pg/mL) | 5.21 | ±0.43 | 8.75 | ±1.46 | **0.025** | 0.053 |
| TNF-α (pg/mL) | 3.64 | ±0.45 | 3.43 | ±0.47 | 0.770 | 0.461 |
| IFN-γ (pg/mL) | 2.71 | ±0.17 | 4.55 | ±0.57 | **0.003** | **0.005** |

Mean ± standard error (SE). *^†^* variables tested following logarithmic transformation. *p*-values of continuous variables were derived from independent *t*-tests; and *^§^* variables were tested by its non-parametric tests (*Mann-Whitney U* tests). All *p*<0.05 were considered to be significant. hs-CRP: high-sensitivity C-reactive protein. GLR: granulocyte to lymphocyte ratio. IFN: interferon. IL: interleukin. MLR: monocyte to lymphocyte ratio. MPR: monocyte to platelet ratio. PLR: platelet to lymphocyte ratio. TNF: tumor necrosis factor. WBC: white blood cell.
